# Intraosseous epidermoid cysts of adjacent digits in a dog

**DOI:** 10.1186/s12917-020-02545-7

**Published:** 2020-09-02

**Authors:** Michail Vagias, Joseph P. Cassidy, Cliona Skelly, Ronan A. Mullins

**Affiliations:** 1 Section of Veterinary Clinical Sciences, University College Dublin, Belfield, Dublin 4, Ireland; 2 Department of Veterinary Pathobiology, School of Veterinary Medicine, Belfield, Dublin 4, Ireland; 3 Equine Clinical Studies, Diagnostic Imaging and Anaesthesia, School of Veterinary Medicine, University College Dublin, Dublin, Ireland

**Keywords:** Intraosseous, Epidermoid, Cyst, Dog, Immunohistochemistry, Digit

## Abstract

**Background:**

Intraosseous epidermoid cyst (IEC) is a rare, non-neoplastic, pathology in animals and humans that most commonly affects the distal phalanx. In dogs, it is important to differentiate this lesion from malignant digital tumours causing bone lysis. In previous reports, IEC has been described to affect only a single digit at the time of diagnosis which is usually based on histopathology. This is the first case report to describe immunohistochemically confirmed IECs affecting simultaneously multiple digits.

**Case presentation:**

A 4-and-a-half-year-old female spayed Great Dane was presented with a 2-month history of progressive swelling of the distal phalanx (PIII) of digits IV and V of the right pelvic limb. Eleven weeks prior to presentation, the dog had a low-grade cutaneous mast cell tumour completely excised from the craniolateral base of its left pinna. A history of trauma to 1 of the nails of the same pes 4 years prior to referral was also reported. Examination of the right pelvic limb identified firm non-painful swelling of PIII of digits IV and V, with concurrent deformation of the nails. Radiographs of the right pes obtained by the primary veterinarian identified an expansile lesion of PIII of digits IV and V. Computed tomography identified large expansile lesions of PIII of digits IV and V, with associated cortical thinning and soft tissue swelling. Neoplasia was considered the most likely radiographic diagnosis. Histopathology of Jamshidi bone biopsies was consistent with intraosseous epidermoid cyst, which was confirmed with immunohistochemistry. Amputation of PIII of digits IV and V at the level of mid-PII was performed as definitive treatment. No recurrence of the lesion occurred during the 10-month follow-up period.

**Conclusions:**

Intraosseous epidermoid cysts should be included in the differential diagnosis for expansile lesions affecting the canine digit. It is important to differentiate them from other digital lesions, with bone involvement, such as malignant digital tumours, which often require more extensive surgery for definitive treatment. The case herein highlights that this lesion can affect simultaneously multiple digits. Definitive diagnosis can be achieved by identification of keratin-producing epithelial cells on histopathology and confirmed by pancytokeratin labelling.

## Background

Intraosseous epidermoid cyst (IEC) is a rare pathology in humans and small animals. Distal phalangeal (PIII) IECs have previously been described in 7 dogs and 2 horses in the English-language veterinary literature [1–6]. There is also a report of an IEC affecting the 10^th^ thoracic vertebra in a dog and the mandible in a horse [4, 7]. In people, the phalanges of the hand and foot are a predilection site [8–22]; however, other reported locations include the distal radius [23], distal femur [24], proximal tibia [25], metacarpal bone [26], frontal and parietal bones of the skull [14, 27–29], styloid process of the temporal bone [30] and maxilla [31]. In all previous reports in animals and humans, simultaneous involvement of multiple digits has not been reported.

Outside of the English-language veterinary literature, there is 1 report describing IECs of the PIII in dogs [32]. In that report [32], the average age of dogs was 10.8 years. A 2.4:1 male-female ratio was reported; however, no breed predispositions were identified. Of the 7 dogs with digital IECs reported in the English-language literature, 3 were female, 1 was male and the sex was not recorded in the remaining 3 dogs [3–6]. Age of affected dogs ranged from 3.5 to 12.0 years [3–5]. In the human literature, phalangeal IECs have been diagnosed in people from the age of 19.0 to 54.0 years and occur more commonly in men than women [8, 11, 20, 33].

The aetiopathogenesis of IECs is unclear but 2 theories have been proposed [9, 10]. The first is related to traumatic implantation of epidermis into bone and the second theory suggests a congenital aetiology [1, 9, 10, 14, 20].

The most common clinical signs reported in the veterinary and human literature for phalangeal IECs include painful swelling of the affected phalanx and nail deformation [3, 4, 9–23, 25, 34].

In humans and animals, IECs are radiographically characterised as well-defined expansile radiolucent osseous lesions with distinct thin-walled sclerotic cortical margins [1, 2, 4, 5, 7, 23, 25, 26, 31, 35]. Other imaging modalities that have been employed to diagnose IECs in people and less commonly in animals include computed tomography (CT) and magnetic resonance imaging (MRI) [2, 11–14, 16, 18, 19, 23–25, 27, 29, 30].

Treatment options for IECs that have been reported in dogs include digital amputation and isolated PIII amputation [3–5, 32]. In the human literature, treatment options include curettage of the lesion [8–14, 16–20, 23–26, 31], curettage combined with packing of the bone cavity with autogenous cancellous or synthetic bone graft [11, 13, 18–20, 23–25, 31], *en bloc* excision and amputation [11, 14, 16, 24, 27]. Regardless of the surgical technique, the mainstay of surgical treatment is to ensure complete excision of the cystic wall in order to avoid recurrence [19, 23].

Histopathology is required for definitive diagnosis of IECs [29]. Microscopically, IECs consist of a cavity filled with keratin that is organised into layers, surrounded by a wall of well-differentiated stratified squamous epithelial cells that undergo normal maturation. The cystic wall is surrounded by a layer of fibrous tissue that separates the bone from the cyst [1–5, 7, 8, 10–15, 17, 19–21, 23–27, 29–31, 33]. In some cases, the cystic wall is ruptured within the bone or surrounding soft tissues resulting in the microscopic characteristics of foreign body reaction [4, 7, 20, 33].

The objective of this case report is to report the simultaneous occurrence of multiple digital IECs in a dog, use of immunohistochemistry for supporting the histopathologic diagnosis and to highlight the importance of differentiating this non-neoplastic disease from other lesions such as malignant digital tumours which require more extensive surgery for definitive treatment.

## Case presentation

**Fig. 1:**
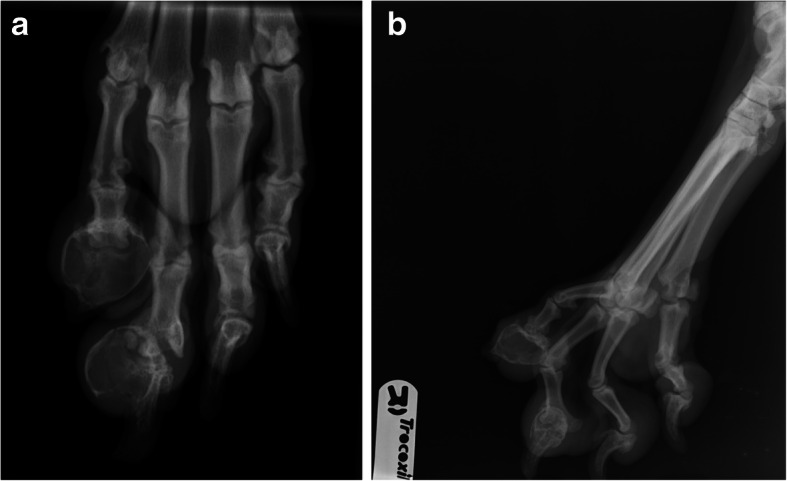
Dorsoplantar (**a**) and splayed mediolateral (**b**) radiographs of the right pes of a 4-and-a-half-year-old 46-kg (101.4-lb) female spayed Great Dane that was presented with a 2-month history of progressive swelling and excessive licking of PIII of digits IV and V of the right pelvic limb. The entire PIII of digit V, and the proximolateral aspect of PIII of digit IV have an expansile, circular, thin-walled osseous cyst-like appearance. There is subluxation of the distal interphalangeal joint of digit IV and new bone formation on the abaxial margins of the distal interphalangeal joints of digits IV and V

A 4-and-a-half-year-old, 46-kg (101.4-lb.), female spayed Great Dane was presented to University College Dublin Veterinary Hospital with a 2-month history of progressive swelling and excessive licking of PIII of digits IV and V of the right pelvic limb. Approximately 11 weeks prior to presentation, the dog had a low-grade cutaneous mast cell tumour excised by the referring veterinarian from the craniolateral base of its left pinna, with clean histologic margins confirmed on histopathology. The dog was presented to its primary veterinarian 2 months prior to referral to our institution for investigation of swelling of digit V of the right pelvic limb, without associated lameness. No wounds were identified in the region of the digital swelling. Trauma to 1 of the nails of the right pelvic limb was reported to have occurred 4 years prior. The dog received a tentative diagnosis of pododermatitis and was prescribed a 2-week course of meloxicam^1^ (0.1 mg/kg, every 24 h [q 24], per os) and amoxicillin clavulanic acid^2^ (14.0 mg/kg, q 24, per os). The dog was re-presented to its primary veterinarian 1 month later and firm, non-painful swelling of PIII of both digits IV and V of the right pelvic limb were identified. A mild decrease in the size of the swelling of digit V was noted. A further 7-day course of meloxicam^1^ (0.1 mg/kg, q 24, per os) and marbofloxacin^3^ (3.5 mg/kg, q 24, per os) was prescribed. Dorsoplantar and splayed mediolateral radiographs of the right pes were obtained by the primary veterinarian 1 week prior to referral to our institution and identified an expansile radiolucent lesion of PIII of digits IV and V. The entire PIII of digit V, including its ungual process, was replaced by a large, expansile, ovoid, thin-walled radiolucent lesion. There was new bone formation on the abaxial margins of the PII-PIII interphalangeal joint. A similar expansile, thin-walled radiolucent lesion, with thin cortices, arising from the lateral aspect of the junction of PIII and its ungual process was identified in digit IV. The ungual process of PIII was present unlike that of digit V. There was subluxation of the PII-PIII interphalangeal joint of digit IV. There was similar new bone formation on the abaxial margins of the PII-PIII interphalangeal joint (Fig. 1a & b). The dog was subsequently referred to our institution for further investigation and treatment.

**Fig. 2:**
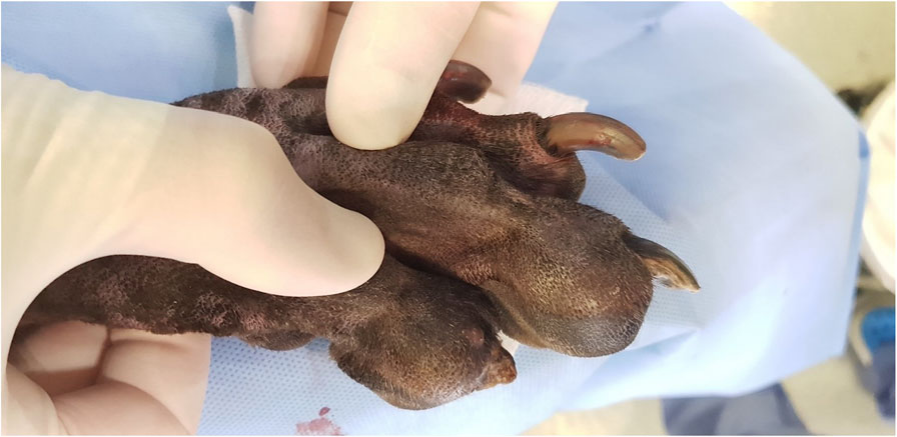
Dorsolateral view of the right pes of the same dog in Fig. 1. A firm, well-defined, swelling of the PIII of digit IV and V causing distortion of the associated nails can be seen

On physical examination, the dog was bright, alert and responsive. Vital parameters were within normal limits. Cardiothoracic auscultation identified a grade II out of VI apical systolic heart murmur, audible only over the left hemithorax. Femoral pulses were strong and synchronous and there were no pulse deficits. Examination of the right pelvic limb identified hard swelling/enlargement of PIII of digits IV and V. There was no apparent pain on deep palpation of affected phalanges or on manipulation of the PII-PIII interphalangeal joints of affected digits. The nail of digit V was abnormally short and conical in shape (Fig. 2). The nail of digit IV was normal in length but was brittle and had a rough surface. The remaining digits were unremarkable. The right popliteal lymph node was moderately enlarged on palpation. Gait assessment identified no lameness.

**Fig. 3:**
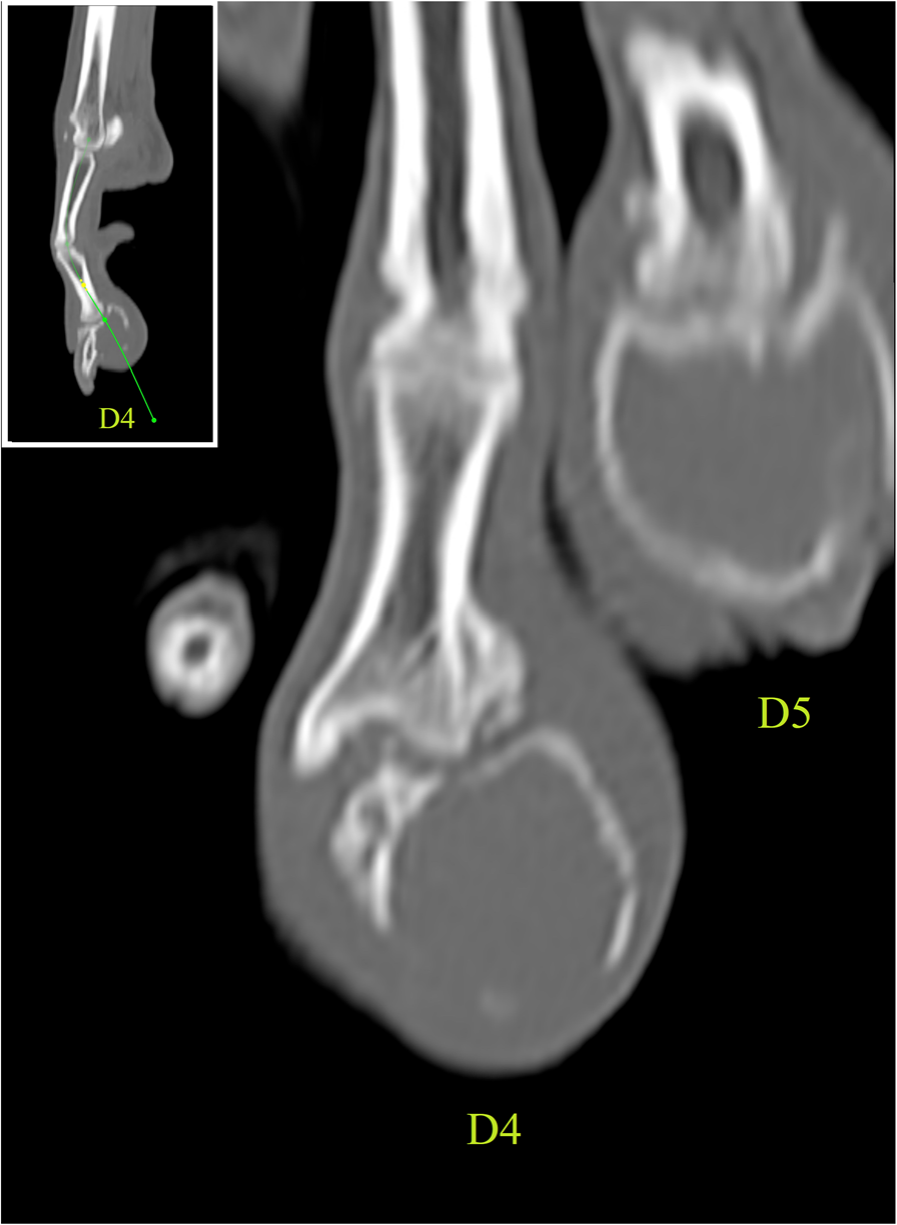
Curved multiplanar reconstruction of the right pes of the same dog in Fig. 1 demonstrating a polyostotic expansile lesion involving the distal phalanx of digit 4 (D4) and digit 5 (D5), with associated cortical thinning and soft tissue swelling. The third phalanx of the digit 4 is moderately laterally subluxated relative to the middle phalanx, with smooth new bone formation extending from and located lateral to the distal metaphysis and epiphysis of the second phalanx of digit 4. Insert: sagittal plane computed tomographic multiplanar reconstruction image of digit 4. The green line indicates the curve used to generate Fig. 3 Window width: 2500, window level: 500.

Complete blood count and serum biochemical analysis identified normal morphology of red and white blood cells, a mild increase in haemoglobin concentration (192 g/L [RI: 120–180]), mild lymphopenia (0.76 × 10^9^/L [RI: 1.00–3.60]), and mild increases in creatinine (123.18 μmol/L [RI: 20.00–120.00]) and alkaline phosphatase (191 U/L [RI: 0–82]). Echocardiography identified a normal left atrium:aortic ratio, normal left ventricular fractional shortening and no evidence of dilated cardiomyopathy. The dog was premedicated with methadone hydrochloride^4^ (0.2 mg/kg), acepromazine maleate^5^ (2.5 μg/kg) and chlorphenamine maleate^6^ (10.0 mg/dog) intravenously, and induced with propofol^7^ (3.5 mg/kg) and ketamine hydrochloride^8^ (2.0 mg/kg) intravenously. Pre- and post-contrast-enhanced CT of the left and right pedes and mani, thorax and abdomen was performed with administration of 600 mgI/kg ioversol^9^ (300 mgI/ml). Large, expansile, radiolucent lesions of PIII of digits IV and V of the right pes were noted, with associated cortical thinning and soft tissue swelling (Fig. 3). A small cortical defect was present on the lateral aspect of the lesion on digit V. The right popliteal lymph node was moderately enlarged. There was no evidence of distant metastasis on thoracic and abdominal CT. Fine needle aspirates of the right popliteal lymph node were obtained for cytologic analysis and were consistent with a normal lymph node. The right pes was clipped of hair and aseptically prepared for surgery. A ring block with lidocaine hydrochloride^10^ (2 mg/kg) was performed. A 2-cm incision was made over the lateral aspect of the PII-III interphalangeal joint of digit V. Two 8-gauge Jamshidi^11^ bone biopsies were obtained from the lateral cortex of PIII and submitted for bacterial and fungal culture and histopathologic analysis. During biopsy acquisition, the Jamshidi^11^ needle accidentally exited the opposite cortex of PIII and entered the interdigital skin. An intraoperative intravenous dose of cefuroxime^12^ (10 mg/kg) was administered following biopsy acquisition. Arthrocentesis of the PII-PIII interphalangeal joint was performed using a 25-gauge hypodermic needle and yielded a very small volume of grossly normal clear to slightly straw-coloured synovial fluid. Cytologic analysis of the synovial fluid identified mild mononuclear inflammation without evidence of infection. The subcutaneous layer was closed with a simple continuous pattern using 3–0 poliglecaprone 25^13^ and the skin with an interrupted cruciate pattern using 2–0 nylon^14^. Immediate postoperative radiographs confirmed optimal location of bone biopsy sites and absence of iatrogenic fracture of PIII. Postoperative analgesia consisted of 2 doses of buprenorphine hydrochloride^15^ (15.0 μg/kg, q 8, intravenously) and acetaminophen^16^ (10.0 mg/kg, q 12, per os) for 4 days. The dog was hospitalized for a total of 2 days postoperatively. Discharge instructions included exercise restriction for 2 weeks, maintenance of an Elizabethan collar, daily observation of the wound and suture removal at 14 days postoperatively. Microbiologic culture of a bone biopsy specimen yielded a heavy growth of a broadly susceptible *Staphylococcus simulans* and a very slow growing *Granulicatella elegans*. No fungal growth was identified. Histopathologic analysis of the 2 Jamshidi^11^ bone biopsies identified small foci of dense lamellar bone with frequent rows of plump osteoblasts lining internal surfaces. Focally within cavities in the bone were small haphazardly-layered keratin squames surrounded by a thin layer of stratified squamous cells (keratinocytes). On IHC, these cells stained positively for pancytokeratin.

The dog was re-presented 7 weeks following initial presentation to our institution for definitive surgery. It was not receiving any medication and demonstrated no lameness. On physical examination, the right popliteal lymph node remained moderately enlarged and firm on palpation. The firm, non-painful, bony swelling of digits IV and V remained static. Fine needle aspirates of the right popliteal lymph node were repeated and cytologic analysis was consistent with a reactive lymph node. The dog was re-anaesthetised and the right crus and pes prepared for surgery as previously described. Intravenous regional anaesthesia was performed using lidocaine hydrochloride^10^ (2.0 mg/kg) via a cannula placed in a dorsal pedal vein after placement of a tourniquet proximal to the tarsocrural joint. The tourniquet was removed intraoperatively after 90 min. A skin incision was made over the dorsal aspect of PIII of digits IV and V extending from the region of the nail bed to the level of mid-PII. The skin and subcutaneous tissue were dissected from the bone (PIII), extending abaxially from dorsal to plantar. Amputation was performed using bone cutters at the level of mid-PII. The ostectomy site was rasped to remove sharp bone edges. During dissection around PIII of digit IV, pale brown-coloured flocculent fluid emanated from an approximately 5–6 mm diameter subungual circular defect in the lesion. A swab of this fluid was obtained for microbiologic culture. Both surgical sites were copiously lavaged with sterile saline. The digital pads of digit IV and V were preserved for closure, which consisted of a simple interrupted suture pattern with 2–0 polydioxanone^17^ in the subcutaneous tissue and an interrupted cruciate pattern with 2–0 polypropylene^18^ in the skin. Postoperative analgesia consisted of a single dose of methadone hydrochloride^4^ (0.1 mg/kg intravenously), and acetaminophen^16^ (10.0 mg/kg, q 12, per os) and meloxicam^1^ (0.1 mg/kg, q 24, per os) for 7 days. Postoperative radiographs of the right pes confirmed complete radiographic excision of both digital lesions. The limb was bandaged postoperatively to protect the surgical sites and was changed q 24 for the first 2 days, at which time the dog was discharged. Discharge instructions included exercise restriction for a further 2 weeks, maintenance of an Elizabethan collar, twice weekly bandage changes, suture removal at 14 days postoperatively and administration of meloxicam^1^ (0.1 mg/kg, q 24, per os) and acetaminophen^16^ (10.0 mg/kg, q 12, per os) for 7 days as well as amoxicillin clavulanic acid^19^ (20.0 mg/kg, q 12, per os) for 14 days.

**Fig. 4:**
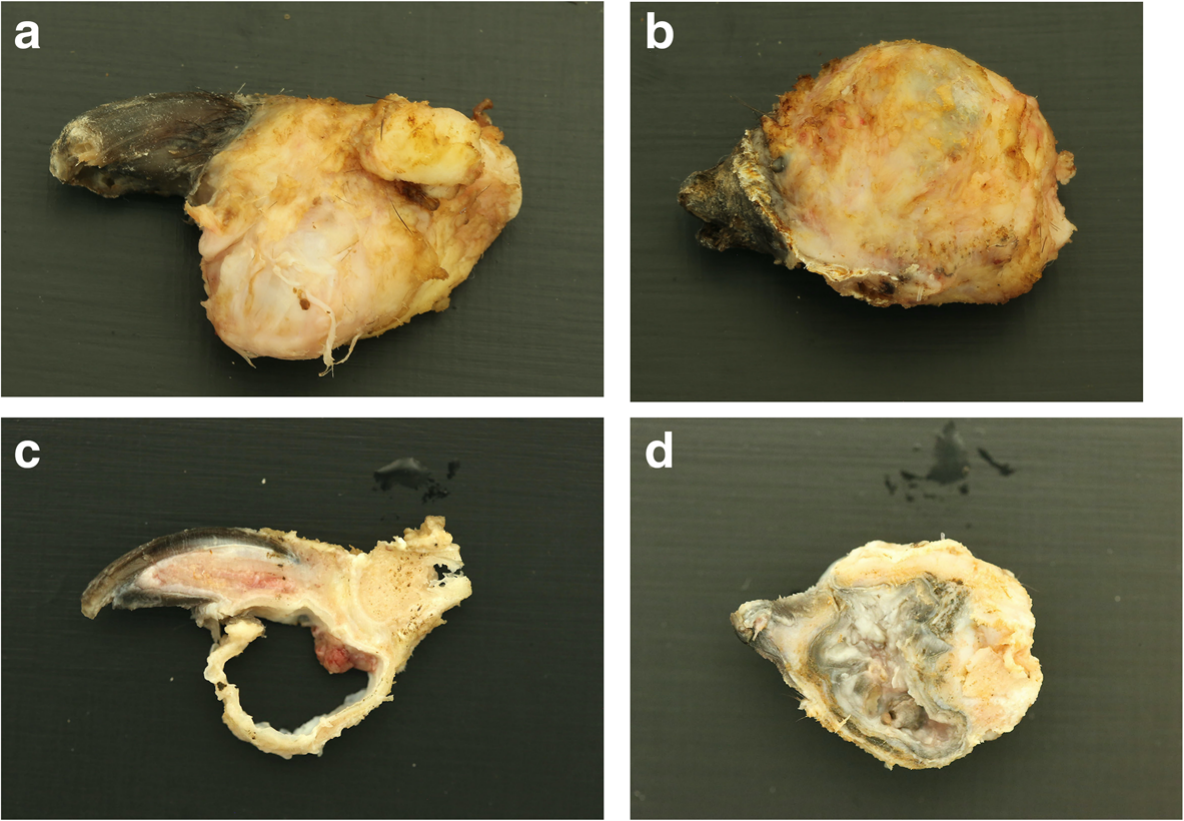
**a**, **b**, **c** & **d** Macroscopic appearance (lateral projections) of amputated distal phalanx of digit IV (**a**) and V (**b**) of the same dog in Fig. 1 after removal of skin and subcutaneous structures. Sagittal (**c**) and dorsal (**d**) cuts of PIII of digits IV and V, respectively. Pale white/grey dense keratin fills the cavity of the cyst (**d**).

**Fig. 5:**
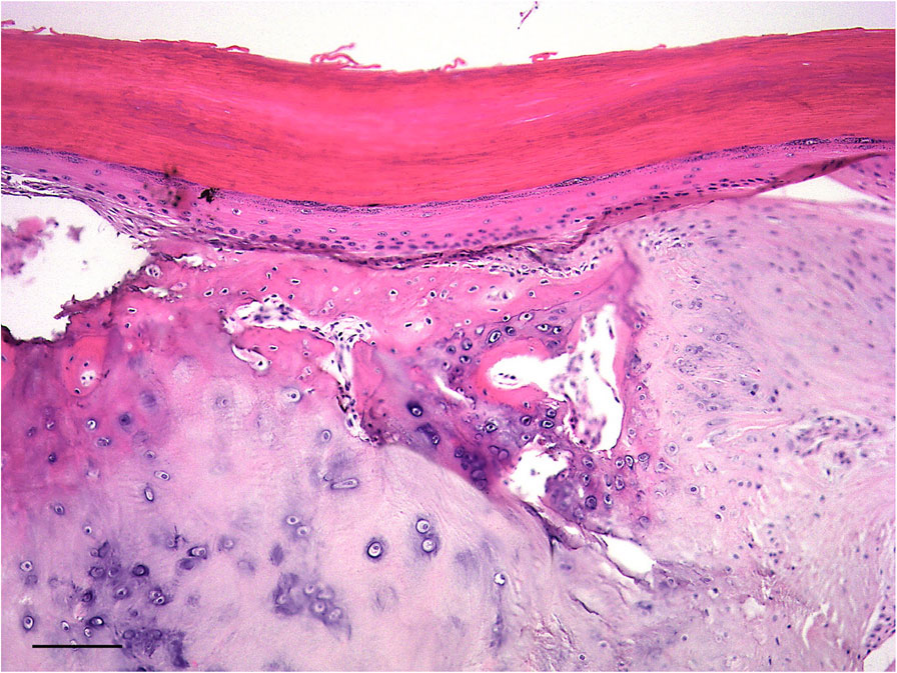
Photomicrograph of excisional biopsy illustrating a cavity (lumen at the top of image) within dense bone lined by thick, gradually keratinizing stratified squamous epithelium with abundant concentrically layered surface keratin. (H&E stain, scale bar = 100 μm)

**Fig. 6:**
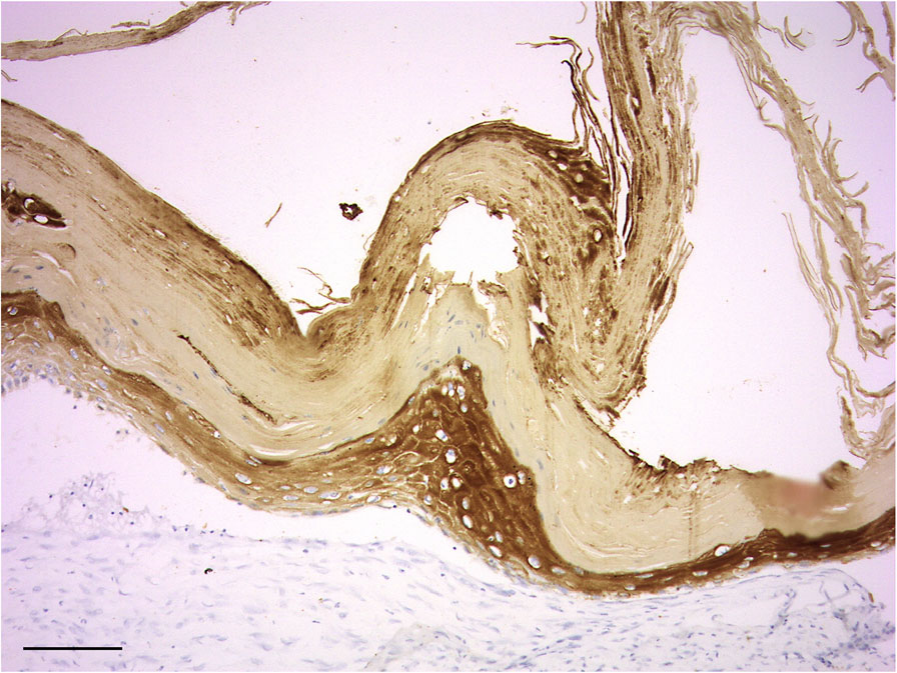
Photomicrograph of the same excisional biopsy in Fig. 5 labelled using pancytokeratin antibody to confirm the epithelial nature of the lining cells

Culture of the fluid emanated intraoperatively from the subungual defect of digit IV yielded no bacterial or fungal growth. The excised phalanges were submitted for histopathologic analysis. Macroscopically, the excised lesions appeared unilocular with pale white/grey dense material filling the cavity (Fig. 4a, b, c & d). Microscopically, a large uniloculated, unencapsulated lesion within dense lamellar bone, deep to a heavily keratinized skin surface was identified. The cavity was lined by a thick, gradually keratinizing stratified squamous epithelium and the lumen was filled with concentric layers of abundant laminated keratin. A layer of mature collagenous, well-vascularised stroma separated the epidermal lining from the surrounding bone. Rows of plump osteoblasts lined bone surfaces (Fig. 5). On IHC, the lining of the cyst stained positive for pancytokeratin confirming the diagnosis of IEC (Fig. 6).

Four days post-discharge, the dog was presented to its primary veterinary clinic for a bandage change and wound check. Dehiscence of both digital pads was identified, which required debridement and open wound management, with daily wound lavage and bandage changes. *Pseudomonas* infection was identified on repeat culture and was treated with marbofloxacin^3^ and amoxicillin clavulanic acid^2^.

## Discussion and conclusions

To the best of our knowledge, this is the first report of an immunohistochemically-confirmed IEC in the veterinary and human literature and the first to describe the simultaneous involvement of multiple digits in the dog and in humans.

In a study involving 17 dogs with digital IECs [32], the thoracic limbs were more commonly affected than the pelvic limbs. In the same study [32], digits IV and V were most commonly affected, which is in agreement with the dog of the present report. Conversely, involvement of multiple digits was not reported in any dog in that study [32]. Furthermore, IHC was not performed in any dog to confirm the suspected diagnosis. In people, manual labourers are more commonly affected and IECs located in the phalanges of the hand with only 1 cyst located in the PIII of the great toe [8, 11, 20].

A history of trauma to 1 of the nails of the affected right pelvic limb was reported in the dog of the present report. The significance of this is uncertain as it is possible that such trauma was completely unrelated to IEC formation. There are several reports of IECs affecting the phalanges of people with a history of previous blunt, penetrating or iatrogenic (e.g. previous surgery) trauma, supporting the traumatic theory for development of IECs [8, 11–22, 24–26, 34–36]. The time interval from such trauma until cysts become clinically evident varies from a few weeks to years [8, 11–22, 24–26, 35]. In humans, the distal phalanges are predisposed to IECs because the skin is in close contact with the bone and the subungual bed adheres to the underlying periosteum in this location [13, 20]. This is similar in dogs, wherein the periosteum of PIII and the dermis of the claw are continuous [37]. The second theory for development of IECs in humans suggests a congenital aetiology, particularly for those located in the skull. It is speculated that during embryologic development, ectoderm becomes entrapped within bone [10, 27, 30, 31]. For the majority of IECs located in the skull, no history of trauma is reported; however, it possible that some patients were unable to recall minor historical injuries [30, 31, 33].

Clinical signs of the case reported herein are similar to those previously described in dogs and in humans, and include firm bony enlargement of the PIII of the affected digits and nail deformation [3, 4, 9–23, 25, 34]. There was no associated pain during physical examination or lameness on gait analysis. In the majority of reports of canine digital IECs, it is not clearly described whether these lesions are associated with pain or lameness [4, 6, 32]. Absence of associated pain on palpation of a PIII lesion similar to the dog of the present report has been previously described [5]; however, in other canine and equine cases, digital IECs are described to cause lameness [1–3]. In people, digital IECs are predominantly painful [9–22, 33–36]. Similarly, IECs affecting other locations such as the femur [24], radius [23], tibia [25] and fifth metacarpal bone [26], are also reported to be painful in humans, whereas other IECs affecting the skull, maxilla and distal phalanx are reported as non-painful [14, 27–29, 31].

Intraosseous epidermoid cysts in both humans and animals share common radiographic characteristics appearing as well-defined expansile radiolucent osseous lesions with distinct thin-walled sclerotic cortical margins [1, 2, 4, 5, 7, 23, 25, 26, 31, 35]. This cortical thinning, which can be observed as the cyst gradually increases in size, can result in fracture or complete bone destruction [10, 11, 13, 19–22, 34]. In the dog described herein, severe cortical thinning associated with the expansile nature of this cystic lesion was observed in both affected phalanges. Fracture of these thin cortices has been reported in humans, which may alter the classic ovoid appearance of IECs [20]. Similarly, in the dog, fracture and subsequent remodelling may alter the characteristic expansile, radiolucent appearance of this lesion [3]. Soft tissue swelling can be also observed surrounding IECs [3, 35], similar to that observed in our case. Based on the CT images in the dog of the present report, a neoplastic process with an atypical appearance was considered the most likely radiologic diagnosis; however, other differential diagnoses including tumour metastases, bone cysts, osteomyelitis and pododermatitis were considered. Prevalence of neoplasia in amputated digits submitted for histopathologic examination is reported as high as 73.3%, with malignant neoplasms comprising 77.7% of all neoplastic lesions [38]. Tumour metastasis was considered a differential diagnosis particularly on the basis of the recently excised mast cell tumour from the left pinna. While there are reports of bone cysts in dogs causing expansile osteolysis with cortical thinning, such lesions are relatively rare and typically affect long bones and were considered less likely than a neoplastic process in this case [39, 40]. A significant association between radiographic evidence of digital lysis and the presence of malignancy has been identified; however, osteolysis can also be observed in cases of pododermatitis and benign neoplasms [41]. It should be noted that the typical lysis associated with malignancies and osteomyelitis tends to be more poorly-defined and destructive in contrast to the well-defined thin-walled (cortices) expansile lesion that is usually observed in cases of IECs and in the dog of the present report [25, 35]. Periosteal proliferation has also been reported in dogs with digital IECs [3] and was observed in the dog of the present report. In the present case, this was isolated to the distal interphalangeal joint and suspected to have been secondary to subluxation of the joint. Simultaneous involvement of multiple digits has not been reported in previous cases of IECs in humans or animals. Multiple digital squamous cell carcinoma have also been reported previously [6, 38, 42]. However, it is not clearly stated in these studies whether multiple digital neoplasia was associated with multiple digital osteolytic lesions [38, 42]. Multiple digital osteolytic lesions have previously been reported in a dog with concurrent osteosarcoma and hemangiosarcoma of the same pes [41]. In a case report by Headley et al., 2 histologically confirmed IECs were described arising from the same PIII of a mare [2]. However, to the authors’ knowledge, IECs simultaneously affecting multiple digits has not been previously reported in the dog.

In the dog described herein, CT of the affected pes was performed in an effort to better define the nature and extent of the osseous lesions identified on radiographs obtained by the primary veterinarian. The main CT features of the dog of the present report included a large expansile lesion with associated cortical thinning and focal cortical loss. We are unaware of other reports describing the use of CT to diagnose IECs in small animals. In people, both CT and MRI are used to diagnose IECs [11–13, 16, 18, 19, 23–25, 27–30]. Typical CT features of digital IECs in people include hypoattenuating, expansile osseous lesions with cortical thinning and destruction [14, 16, 23, 25, 27, 29, 30]. To the authors’ knowledge MRI has not been reported in the veterinary literature for the diagnosis of IECs.

Macroscopically, IECs lesions tend to be spherical, filled with thick semi-liquid, light-coloured cream-to-gray-tinged material [1–4, 7–9, 12, 14–24, 27, 31, 33, 34]. In the dog of the present report, thick, pale brown, flocculent material emanated from the cystic structure during dissection, similar to what has been described previously.

Cytology of fine needle aspirates has been described as a minimally invasive method of obtaining a diagnosis in human patients but has not been described in the veterinary literature [16, 17, 22]. Fine needle aspirates could have been obtained as an initial diagnostic step and a less invasive alternative to obtaining Jamshidi^11^ bone biopsy specimens in the dog of the present report. However, on the basis of the possibility of a non-diagnostic sample, histopathology of surgically obtained bone biopsy specimens was considered more likely to provide a definitive diagnosis. Being epithelial in origin, IECs exfoliate well, with the most common cytologic findings including anucleate squames surrounded by granular debris [17, 22]. The gross characteristics of the aspirate can also support the diagnosis, with several investigators in the human literature reporting thick, white, semi-liquid material [17, 19, 22].

Secondary infection of IECs is infrequently reported in both the veterinary and human literature [1, 23]. In 1 report of an IEC in the PIII of a horse and another in the distal radius of a woman, *Pseudomonas aeroginosa* and *Staphylococcus aureus* were isolated at the time of surgery, respectively [1, 23]. Initial culture of the IEC in the dog of the present report yielded *Staphylococcus simulans* and *Granulicatella elegans*. However, both were considered to be contaminants due to inadvertent penetration of the interdigital skin during biopsy acquisition. In the human literature, the majority of IECs are sterile [10, 15, 16, 24, 26, 36]. It is likely that people will seek medical attention much earlier in the disease course before a cyst potentially becomes infected. Digital IECs in animals may be more prone to trauma and bacterial contamination/infection as they are quadrupeds and for those that occur in the pelvic limbs do not have the protection offered by footwear as is the case in people.

Treatment options that were discussed for the dog of the present report included curettage and autogenous cancellous bone grafting, mid-PII amputation and isolated PIII excision (declawing). Partial foot amputation has been reported in dogs for the management of malignant tumours of the digits; however, in light of the histopathologic diagnosis of benign IEC and the distal location of the lesion within the digits, this option was not considered appropriate. To the authors’ knowledge, curettage and bone grafting as is reported in people has not been described for the management of IECs in dogs [8, 19, 23, 24]. Amputation at the level of mid-PII was discussed with the owner as an alternative to isolated PIII excision (declawing) on the basis that the articular cartilage is more richly innervated and contact of the cartilage of the distal PII with the ground during weight bearing may be more likely to result in lameness [43]. On the basis of the paucity of information describing outcomes following curettage and bone grafting of IECs in dogs, the risk of bone fracture due to the severity of cortical thinning in our case and the risk of recurrence with incomplete excision, the owner elected for mid-PII amputation. While amputation of the affected digits at the level of the mid-PII was elected by the owner as the definitive treatment in the dog of the present report, curettage and bone grafting or isolated PIII excision would likely have been equally appropriate. Curettage of the IECs is frequently performed in people and care is advised to ensure complete excision of the cystic wall is achieved [8, 19, 23, 24]. Bone grafting after curettage is controversial, with some investigators recommending grafting when cortical thinning is present and pathologic fracture is a concern [19]. In people, IECs located in the skull, those that result in severe bone destruction and those that recur after curettage are treated more aggressively with *en bloc* excision or amputation of affected phalanges [11, 14, 16, 24, 27, 34, 36]. Recurrence of IECs in people is usually associated with incomplete excision of the cystic wall and has been reported within 5 months to 9 years postoperatively [14, 16, 19, 23, 29]. No recurrence was reported in previously published cases of IECs in dogs treated with digital amputation [3, 5] or isolated PIII amputation [4]. No recurrence was identified in the dog of the present report during the 10-month follow-up period.

The use of IHC has not been previously described in veterinary and human literature in order to support the histopathologic diagnosis of IECs. In the case described herein, pancytocekeratin labelling was used on the initial Jamshidi^11^ bone biopsies, as definitive diagnosis was uncertain based on the histopathology. Pancytokeratin labelling confirmed the epithelial nature of the lining cells of the cyst further supporting the histopathological suspicion of IEC, which allowed the authors to consider less aggressive treatment options. Repeat IHC was performed after excision of the lesion to confirm the initial diagnosis.

In conclusion, IECs are a rare pathology in dogs but should be considered as differential diagnosis for expansile digital lesions. It is important to differentiate them from other digital lesions, with bone involvement, such as malignant digital tumours, which often require more extensive surgery for definitive treatment. We believe that knowledge of the characteristic expansile thin-walled radiologic nature of this lesion in a known predilection site (distal phalanx) in dogs and in humans may expedite definitive treatment and potentially avoid the requirement for incisional biopsy as was required in the case herein. This report highlights that IECs can affect simultaneously multiple digits. Definitive diagnosis can be achieved with histopathology supported by immunohistochemical labelling for pancytokeratin.

## Data Availability

All data generated or analysed during this study are included in this published article.

## Abbreviations

IEC: Intraosseous epidermoid cyst
IHC: Immunohistochemistry
PII: Second phalanx
PIII: Third phalanx
RI: Reference interval

## Footnotes

1 Metacam; Boehringer Ingelheim Vetmedica GmbH, Ingelheim, Germany

2 Kesium; Ceva Animal Health Ltd., Buckinghamshire, UK

3 Marbocyl; Vetoquinol Ireland Ltd., Dublin 2, Ireland

4 Synthadon; Animalcare Ltd., York, UK

5 Acecare; Duggan Veterinary Supplies Ltd., Tipperary, Ireland

6 Chlorphenamine; KYOWA KIRIN Ltd., Galashiels, UK

7 Propofol-Lipuro; B. Braun, Melsungen, Germany

8 Ketamidor; Chanelle Pharmaceuticals Manufacturing Ltd., Galway, Ireland

9 Optiray; Guerbet, Roissy, France

10 Lidocaine Hydrochloride; B. Braum Medical Ltd., Dublin 12, Ireland

11 Jamshidi; CareFusion France 309 S.A.S., Chateaubriant, France

12 Zinacef; GlaxoSmithKline Ireland Ltd., Dublin 24, Ireland

13 Monocryl; Ethicon, USA

14 Ethilon; Ethicon, USA

15 Buprecare; Animalcare Ltd., York, UK

16 Paralief; Clonmel Healthcare Ltd., Clonmel, Ireland

17 PDS II; Ethicon, USA

18 Prolene; Ethicon, USA

19 Noroclav; Norbrook Laboratories (Ireland) Limited, Monaghan, Ireland
